# Removal of Acidic-Sulfur-Containing Components from Gasoline Fractions and Their Simulated Analogues Using Silica Gel Modified with Transition-Metal Carboxylates

**DOI:** 10.1021/acsomega.1c02777

**Published:** 2021-08-27

**Authors:** Andrey O. Okhlobystin, Igor L. Eremenko, Valentina N. Storozhenko, Kseniya V. Oleinikova, Anna S. Kamyshnikova, Konstantin P. Pashchenko, Elena V. Shinkar’, Ekaterina N. Zorina-Tikhonova, Mikhail A. Kiskin, Alexander E. Baranchikov, Sergey Yu. Kottsov, Nadezhda T. Berberova

**Affiliations:** † Department of Chemistry, Astrakhan State Technical University, Astrakhan 414056, Russia; ‡ Department of Chemistry of Coordination Polynuclear Compounds, N.S. Kurnakov’s Institute of General and Inorganic Chemistry of the Russian Academy of Sciences, Moscow 119991, Russia

## Abstract

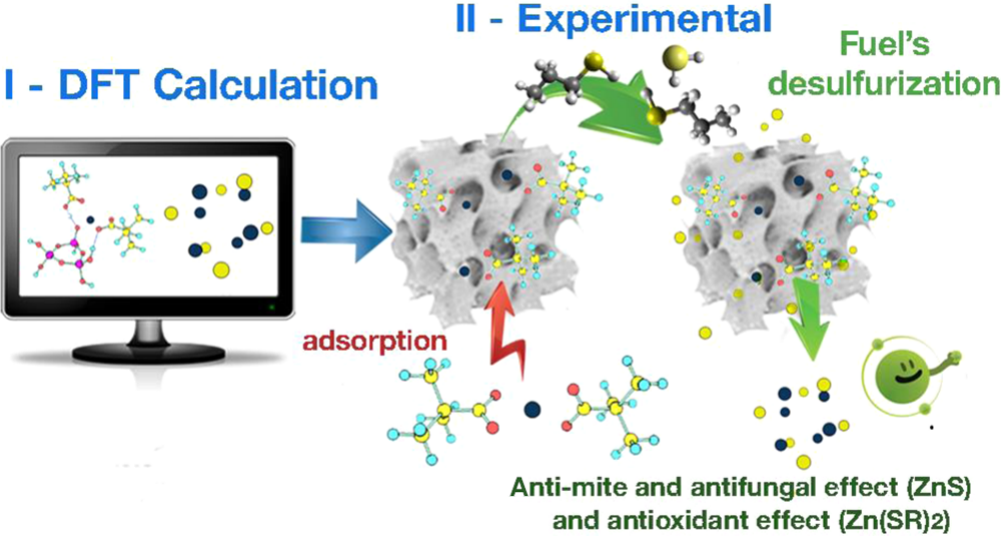

The removal of acidic sulfur-containing components [hydrogen sulfide (H_2_S) and alkanethiols or thiols (RSH)] from simulated mixtures and analogues of gasoline fractions with Zn(II), Cu(II), Co(II), and Ni(II) acetates, pivalates, and malonates applied on silica gel with various porosities under ultrasonic treatment in solution has been studied. The dependence of the adsorption of H_2_S and RSH on the surface of silica gel modified by metal complexes with organic ligands on various factors (the pore size of the silica gel, the time of ultrasonic treatment, and the nature of carboxylate complexes) is established. The best results for the removal of total sulfur from the model mixture and an analogue of the gasoline fraction were obtained using silica gel modified with zinc pivalate (96%) and cobalt pivalate (95%). A waste-free method to desulfurize fuel with zinc pivalate based on the production of practically useful ZnS is suggested.

## Introduction

Lately, oxidation methods have been increasingly used to remove hydrogen sulfide (H_2_S) and low-molecular-weight thiols (RSH) from hydrocarbon fuels in the presence of catalytic amounts of complexes of transition metals that enable desulfurization under energy-favorable standard conditions. Corrosive, highly toxic H_2_S and RSH are contained in light straight-run fractions, fuel oil, and residual products of secondary treatment of high-sulfur hydrocarbon raw materials or gas condensates. Commercial hydrotreatment of light distillates is rather efficient but at the same time highly power- and hydrogen-consuming. Hydrotreatment enables a significant reduction in the content of sulfides; however, in some cases, fuels should be additionally treated to attain standard parameters with application of energy-saving and environment-friendly methods. It should be noted, however, that the search for new types of agents for absorption/adsorption of acidic sulfur-containing components that cause corrosion and deactivation of catalysts remains of importance to date.

Since low-molecular-weight RSH feature weaker acidity than H_2_S, the efficiency of nitrogen-containing organic compounds for RSH absorption is insufficient. Adsorption methods are widely used for desulfurization. Attention in modern publications is focused on mesoporous materials and their composites that are used as adsorbents of H_2_S and RSH.^1−7^ Although mesoporous adsorbents have many beneficial features, their low commercial availability should be taken into account.

Modifying the surface of porous materials with organic or inorganic functional groups enables the physicochemical properties of the adsorbent and the reactivity of the surface layer to be varied. Formulations that contain salts of transition metals (Zn, Fe, etc.) and water-soluble aldehydes,^8^ as well as carboxylates of these metals (acetates and formates),^9,10^ exhibit a positive effect when used as modifiers of porous carriers in the removal of H_2_S and low-molecular-weight RSH from gas mixtures. We have shown earlier^11−13^ that complexes of transition metals (Ni, Cr, Pt, and Pd) with quinoid-type redox ligands facilitate the oxidative activation of H_2_S and RSH to the corresponding cation radicals. Subsequent dimerization of sulfur-centered radicals results in polysulfanes from H_2_S and symmetric dialkyl disulfides [M(RS)_2_] from RSH. These redox catalysts can be regenerated on treatment with atmospheric oxygen. It is, however, inexpedient to use these complexes as modifiers of porous carriers due to the large number of steps involved in the synthesis of these compounds and the inevitable losses upon several stages of adsorption.

To remove H_2_S from gases and fuels, composite adsorbents have been suggested comprising metal oxides and porous adsorbents such as activated carbon,^14−19^ mesoporous silica,^20^ and zeolites.^21,22^ These porous adsorbents feature a high surface area and a large volume of pores that ensure high adsorption capacity, while metal oxides react with H_2_S to produce metal sulfides.

For example, a synthesis of a series of bifunctional ZnO/MgO-activated carbon adsorbents intended for H_2_S removal at ambient temperature in dynamic anaerobic conditions was reported.^23^ The bifunctional adsorbents were synthesized by impregnation with an aqueous solution of Zn(NO_3_)_2_·6H_2_O and Mg(NO_3_)_2_·6H_2_O with various molar Zn/Mg ratios followed by drying at 30 °C and calcination in a nitrogen atmosphere at 350 °C. The phase composition of the surface before and after adsorption was studied using X-ray diffraction analysis. Nitrates were proved to fully decompose to metal oxides upon calcination. Analysis of the adsorption isotherms showed that the deposited metal oxides reduced the porosity of the adsorbent. It was found that as the molar fraction of MgO increases, the surface area and porosity of the adsorbent decrease due to blocking of micropores with MgO while the mesoporosity factor increases. Analysis of X-ray diffraction patterns of the surface of adsorbents after adsorption shows that a reaction occurs between ZnO and H_2_S, while MgO and H_2_S do not react at room temperature. Spectral methods were used to detect the formation of ZnS, sulfur, and sulfates upon H_2_S removal. The presence of sulfates is explained by catalytic oxidation of H_2_S. By varying the molar ratio of magnesium and zinc oxides, a high extent of H_2_S removal (113.4 mg/g) by the adsorbent was attained. Such an efficiency of H_2_S removal is due to MgO basicity. The addition of MgO along with ZnO increases the adsorbent basicity and facilitates the dissociation of H_2_S, which enhances the chemical adsorption on the active phase of ZnO and catalytic oxidation of H_2_S to elemental sulfur. It has been shown earlier that the oxidation of H_2_S primarily occurs in micropores, while the chemical adsorption occurs in mesopores.^24,25^

The efficiency of a zeolite modified with ZnO for the removal of H_2_S from a gas flow at low temperature was demonstrated.^26^ The adsorbent was prepared by impregnating with a solution of zinc nitrate of certain concentration to obtain 10, 20, and 30 mass % of ZnO on the zeolite, followed by drying at 105 °C and calcination in air at 300 °C. The chemical composition of the oxygen-modified zeolite frames was determined using scanning electron microscopy (SEM). It was shown that the optimal concentration of ZnO was 20 mass %, since a further increase in concentration resulted in the destruction of the zeolite structure. Analysis of the adsorption isotherm after the modification of zeolite by ZnO showed that the surface area and volume of zeolite micropores decreased, which indicated that ZnO was present in the porous zeolite structure and in the vicinity of the outside surface. Based on the adsorption/desorption properties of N_2_, it is assumed that an increase in the concentration of ZnO affects the dimensions of zeolite pores. The zeolite modified with 20 mass % ZnO exhibited the highest adsorption capacity of the adsorbent with respect to H_2_S (15.75 mg/g). A reduction in the adsorption capacity was observed when the content of ZnO exceeded 20 mass %, which is explained by the blocking of zeolite pores.

The adsorption activity of synthesized nanocomposites: ZnO–zeolite Y, CuO–zeolite Y, CeO_2_–zeolite Y, zeolite Y with respect to H_2_S in diesel oil was studied. The zeolite nanocomposites modified with metal oxides were synthesized by impregnation with aqueous solutions of metal acetates at 85–90 °C followed by the addition of 0.2 M NaOH solution. The resulting nanocomposite was dried at 400 °C. The X-ray diffraction method was used to detect the presence of CuO, ZnO, and CeO_2_ in modified zeolite samples. The maximum mean size of zeolite particles determined using SEM was in the range of 101–153 nm. The effect of the type of metal on the size and shape of nanocomposites was studied. The formation of semispherical particles with a mean nanostructure size in the range of 50–72 nm was observed. The catalytic activity of the MeO/zeolite nanocomposites obtained was studied with respect to diesel oil. The results obtained show that the desulfurization activity of nanocomposites in a 5 h continuous process decreased in the series: ZnO–zeolite Y > CuO–zeolite Y > CeO_2_–zeolite Y > zeolite Y. The results of the experiment show that the desulfurization of diesel oil with ZnO–zeolite Y is more thermodynamically advantageous. According to published data,^27^ the negative standard Gibbs free energy (Δ*G* < 0) is an indication that the adsorption of sulfur-containing compounds on ZnO occurs spontaneously and exothermically. The results obtained show that the “ZnO–zeolite Y” combination can be used as a new adsorbent for sulfur removal.

Mixed adsorbents based on highly dispersed ZnO–CuO applied onto commercial activated carbon by impregnating with aqueous solutions of metal nitrates followed by thermal treatment at 250 °C in a flow of an inert gas were obtained.^28^ The concentration of metals (Zn and Cu) in the modified adsorbent was 10 mass %, while the relative content of Zn and Cu was varied, including the adsorbent modified with ZnO or CuO alone.

The adsorption properties of all adsorbents modified with pure or mixed oxides are considerably better than those of unmodified activated carbon. It was shown, in particular, that an adsorbent with equimolar amounts of CuO and ZnO was superior to both analogues that contained 100% Zn or Cu in regards to sulfur saturation capacity and sorption kinetics; an apparent synergistic effect between the two metal oxides was also discovered. The results of the experiment show that Zn and Cu sulfates are produced at a high rate primarily at the initial stage of chemical adsorption.

Zhang et al.^29^ synthesized mesoporous carbon spheres that were modified with MgO. The adsorbent modification included impregnation with an aqueous Mg(NO_3_)_2_·6H_2_O solution followed by calcination at 400 °C and thermal treatment in nitrogen. In addition, adsorbents modified with inorganic salts and alkali were obtained using the impregnation technique but without the thermal treatment. The morphology of the adsorbent is characterized by a spherical shape and smooth surface without defects. It was observed that the adsorbent porosity decreased after impregnation due to the filling of pores with heavier inorganic salts or oxides. Nitrogen adsorption was used to prove that all the specimens maintained significant mesoporous structures with a high volume of pores. The large pore volume can be used for the adsorption of sulfur removal products, thus enhancing the adsorption capacity of adsorbents. Analysis of the adsorption capacity of adsorbents modified with inorganic salts and alkali showed their high capacity for H_2_S removal. The adsorption capacity is 4–6 times higher than that of commercial adsorbents based on activated carbon.^30^ It was hypothesized that H_2_S first dissociates into HS^–^ in a moist environment on a carbon surface and then HS^–^ ions are oxidized by oxygen radicals to elementary sulfur. In addition, the effect of an increase in the MgO concentration from 5 to 20 mass % was analyzed. It was shown that as the MgO concentration increases, the total volume of pores decreases. The results obtained by SEM indicate that Mg is uniformly distributed over the entire adsorbent. The surface chemical composition of a used-up adsorbent was analyzed by SEM and transmission electron microscopy (TEM). The presence of sulfur and Mg on the surface of the used-up adsorbent was confirmed. Thus, the developed adsorbent based on MgO is efficient for H_2_S removal at low temperatures. The characteristics of basic MgO proved to be considerably superior to those of basic salts and alkali, which is due to the difference in their solubility in an aqueous medium during the sorption process.

Carbon nanofibers functionalized with iron were used as an H_2_S adsorbent at 100 °C.^31^ The carbon nanofibers were modified by impregnating with an aqueous solution of Fe(NO_3_)_3_·9H_2_O. The amount of metal in the final product was 20 mass %. As the temperature was increased from 100 to 300 °C, the adsorption capacity with respect to H_2_S also increased.

The synergistic effects in composites of iron oxide (Fe_2_O_3_) with oxygen-functionalized porous carbon (OPC) used for H_2_S removal at room temperature were studied.^32^ Two types of Fe_2_O_3_–OPC composite specimens were produced by mechanical and chemical mixing. The efficiency of both types of composites for the absorption of H_2_S under ambient conditions was tested, and the synergistic effects of Fe_2_O_3_ and OPC were studied. The H_2_S adsorption capacity of the specimens obtained by mechanical mixing of components was inferior to that of pure Fe_2_O_3_ or OPC, which indicates a negative synergistic effect. The composite specimens obtained in a chemical way manifest a positive synergistic effect and exhibit the highest adsorption capacity (95%).

Loading metal oxides or mixed metal oxides into porous adsorbent structures enhances the adsorption capacity with respect to H_2_S even at low temperatures owing to the formation of a strong metal–sulfur bond. However, excessive loading of a metal oxide results in the blocking of pores or aggregation of particles and, consequently, in a reduction in the desulfurization efficiency. Thus, development of more efficient materials that contain large voids or channels for loading active centers without a loss in the adsorbent porosity is of primary importance for attaining a better gas–solid reaction and preventing resistance to H_2_S diffusion. It should be noted that finding a porous adsorbent that features all the required characteristics including a high H_2_S adsorption capacity, significant selectivity, and full regeneration capability is a challenging problem. It should also be taken into account that in most cases, modification of porous adsorbents with metal oxides involves high temperatures.

A high extent of purification of hydrocarbon raw materials from sulfur compounds can be achieved under mild conditions using both water-soluble and oil-soluble chelate metal compounds of Zn(II) or Fe(III).^33^ The chelates contain carboxylate groups derived from nitrilotriacetic acid, ethylenediaminetetraacetic acid (EDTA), polyamino disuccinic acid, or gluconates.^34−37^ Metal sulfides are formed as the desulfurization products. The absorbent thus obtained reacts with H_2_S to give fine dispersions of ZnS or Fe_2_S_3_ in the oil phase as expressed in eq 1
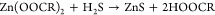
1

If the metal is in a high oxidation state, then H_2_S is oxidized to sulfur.^38,39^

It is stated^40^ that it is possible to use carboxylates (octoates, dodecanoates, naphthenates) of transition metals from the group including Zn, Fe, Co, Ca, Mn, or their mixtures for the removal of H_2_S, mercaptans, sulfides, and other sulfur-containing compounds from oil and hydrocarbon gases. The concentration of the complexes is varied from 10 to 100 mass %. The efficiency of the absorbers is from 71.4 to 98.3%.

It was found^41^ that the concentration of H_2_S in hydrocarbon mixtures could be reduced by adding the oxo complex of zinc carboxylate obtained by the reaction of ZnO particles with a mixture of two or more carboxylic acids (including acetic, oleic, isobutyric, linolenic, and neodecanoic acids), where the oxo complex of zinc carboxylate is soluble in hydrocarbons. Low-molecular-weight acids can be incorporated by synthesizing a complex that is coordinated with a mixture of acid ligands, thereby producing a complex with a lower molecular weight. A decrease in the total molecular mass of the oxo–zinc complex results in a complex with a higher mass content of zinc and increases the profitability of the final product in terms of H_2_S absorption efficiency. The use of low molecular weight acids does not affect the H_2_S removal efficiency of the product. ZnS is formed as the desulfurization product. The purification of a hydrocarbon mixture reduced the content of H_2_S from 200 to 28 ppm in 2 h of treatment and to 22.5 ppm in 24 h of treatment.

Taking the above data into consideration, we studied available transition-metal salts, namely pivalates, derivatives of trimethylacetic acid. Previously, we performed experiments for demercaptanization of diesel oil and simulated mixtures using γ-Al_2_O_3_ modified with nanostructured layers of metal oxides obtained by the calcination of a mixture of aluminum hydroxide and transition-metal pivalates. The adsorbents of a new type produced in this way actively adsorbed acidic sulfur-containing components from fuels: the residual content of H_2_S and mercaptans was less than 10 ppm.^42^

In this work, we studied an environmentally clean energy-saving technique to remove acidic sulfur-containing components (H_2_S and RSH) from a simulated fuel using silica gel coated with metal carboxylates by impregnation under ultrasonic treatment. Coordination compounds of 3d metals with carboxylate ligands feature unusual physicochemical properties. These complexes of transition metals were selected as modifiers of the adsorbent surface due to good solubility and stability in organic solvents, as these features facilitate efficient impregnation of the adsorbent.

The preparation of these H_2_S and RSH adsorbents involved varying a number of conditions: carrier pore size, duration of its ultrasonic treatment during modification with metal salts, height of the filling layer, extent of retention of carboxylates in pores and on the adsorbent surface, and the nature of the anion and metal.

The purpose of this study was to explore the modification of the silica surface with carboxylates (acetates, pivalates, and “malonates”) of transition metals (Zn, Cu, Co, and Ni) under ultrasonic treatment in order to produce adsorbents that would be promising agents for a significant reduction in the content of toxic sulfides in fuels.

## Experimental Section

### Synthesis of Transition-Metal Carboxylates

Standard techniques were used to synthesize pivalates of transition metals (Zn, Cu, Co, and Ni): zinc(II) pivalate [Zn(Piv)_2_]_*n*_, copper(II) pivalate [Cu_2_(Piv)_4_(Hpiv)_2_],^43^ cobalt(II) pivalate [Co(Piv)_2_]_*n*_,^44^ and nickel(II) pivalate [Ni_9_(OH)_6_(Piv)_12_(Hpiv)_4_].^45^ We used zinc(II) acetate dihydrate Zn(OAc)_2_·2H_2_O (“chemically pure”, ECROS, Russia) and cobalt(II) acetate tetrahydrate (“pure for analysis”, ECROS, Russia) as silica gel modifiers.

### Synthesis of Zinc “Malonate”

The compound was obtained using ethanol (96%), acetonitrile (“chemically pure”, Khimmed, Russia), Zn(OAc)_2_·2H_2_O (99%, Roth, Germany), cyclobutane-1,1-dicarboxylic acid (99%, Sigma-Aldrich, Germany), and tetrabutylammonium hydroxide (40% aqueous solution) (“commercially pure”, Sigma-Aldrich, Germany). Zn(OAc)_2_·2H_2_O (0.20 g, 0.91 mmol) was added to a solution of tetrabutylammonium cyclobutane-1,1-dicarboxylate obtained from 2.35 g of 40% aqueous solution of tetrabutylammonium hydroxide (3.64 mmol) and 0.26 g (1.82 mmol) of (cyclobutane-1,1-dicarboxylic acid) in ethanol (20 mL). The reaction mixture was stirred for 1 h with heating (*t* = 50 °C). The resulting colorless solution was concentrated to 10, then 10 mL of acetonitrile was added, and the mixture was kept for 72 h. The precipitated colorless crystals suitable for X-ray single-crystal analysis were filtered off, rinsed with water, and dried in air at *t* = 20 °C. Found (%): C, 61.15; H, 9.98; and N, 3.07. C_88_H_174_Zn_2_N_4_O_19_. Calcd (%): C, 61.34; H, 10.18; and N, 3.25. IR spectrum (attenuated total reflection), ν/cm^–1^: 3400 m, 2990 m, 2968 m, 2950 m, 2866 m, 1616 s, 1567 s, 1468 m, 1430 m, 1360 s, 1259 m, 1224 m, 1161 m, 1120 m, 1011 m, 951 m, 916 m, 856 m, 761 s, 716 s, 667 s, 630 s, 590 s, 559 s, 515 s, 474 s, and 452 s.

### XRD Analysis Results

XRD analysis of a single crystal of (NBu_4_)_4_[Zn(cbdc)_2_]_2_·3H_2_O was performed using a Bruker APEX II (Bruker AXS, Karlsruhe, Germany) diffractometer equipped with a charge-coupled device detector (Mo Kα, λ = 0.71073 Å, graphite monochromator).^46^ A semi-empirical correction factor was introduced to take absorption into account using the SADABS program.^47^ The structure was resolved using the direct method and refined using the least-squares method, first in the isotropic and then in the anisotropic approximation versus *F*_*hkl*_^2^. The positions of hydrogen atoms were calculated geometrically and refined in the isotropic approximation using the riding model. All calculations were carried out using the SHELXL software package.^48^ The crystallographic parameters and structural details are presented in Table 1.

**Table 1:** Crystallographic Parameters and the Refinement Statistics for (NBu_4_)_4_[Zn(cbdc)_2_]_2_·3H_2_O

empirical formula	C_88_H_174_N_4_O_19_Zn_2_
formula weight (g/mol)	1723.04
temperature (K)	296(2)
crystal system	triclinic
space group	*P*1̅
*a* (Å)	11.7640(6)
*b* (Å)	19.9620(10)
*c* (Å)	21.5066(11)
α (deg)	89.0379(8)
β (deg)	87.3767(8)
γ (deg)	89.6663(8)
volume (Å^3^)	5044.4(4)
*Z*	2
calculated density (g/cm^3^)	1.134
adsorption coefficient (mm^–1^)	0.537
reflections collected	54313
reflections unique	21359
reflections with *I* ≥ 2σ(*I*)	12242
*R*(int)	0.029
goodness-of-fit on *F*^2^	1.039
θ range (deg)	1.02–26.73, –14 ≤ *h* ≤ 14, –25 ≤ *k* ≤ 25, –27 ≤ *l* ≤ 27
*T*_min/max_	0.6460/0.7456
*R*_1_/w*R*_2_ (for all data)^a^	0.1275/0.2742
*R*_1_/w*R*_2_ (for *I* ≥ 2σ(*I*))^a^	0.0769/0.2306
Δρ_min_/Δρ_max_	–0.578/1.124 Å^–3^
CCDC deposition number	2051019

a *R*_1_ = Σ||*F*_o_| – |*F*_c_||/Σ|*F*_o_|, w*R*_2_ = {Σ[*w*(*F*_o_^2^ – *F*_c_^2^)^2^]/Σ[*w*(*F*_o_^2^)^2^]}^1/2^.

### Characteristics of the Carrier

To select the optimal amount of silica that ensures the maximal absorption of acidic sulfur-containing components in the simulated fuel, we used silica gel brands from Sigma-Aldrich and Alfa Aesar with pore dimensions of 2.2, 3.0, 6.0, 9.0, and 15.0 nm.

### Determination of the Specific Surface Area

The specific surface area of specimens was measured by low-temperature adsorption of nitrogen using an ATX-06 (Katakon, Russia) device. The specimens were degassed in a flow of N_2_ (1 atm) for 1 h at 200 °C. The specific surface area of specimens was determined using the Brunauer–Emmett–Teller (BET) model by the five-point method in the range of partial pressures varying within 0.05–0.25.

### Preparation of the Adsorbent

Silica gel was modified with Zn(II), Cu(II), Co(II), and Ni(II) carboxylates using the impregnation method under ultrasonic treatment. Silica gel was dried in a vacuum-drying oven for 24 h at 100 °C. To modify the silica gel, 1 mass % solutions of the metal salts in isopropyl alcohol were used. A solution of a modifier and silica gel was placed in an ultrasound bath (generator power, 180 W; operating frequency, 40 kHz) to intensify the diffusion of the carboxylate solution into the porous structure of the carrier. The optimal duration of ultrasonic treatment of the modifier solution in the presence of the carrier is 90 min; longer treatment is unreasonable because the effect attained is insignificant. The silica gel modified in this way was dried in air for 24 h. The adsorbent was kept after removal of the solvent in a vacuum-drying oven for 24 h at a temperature of 100 °C.

### Methods for Monitoring the Content of Metals on the Carrier

The extent of retention of polymeric carboxylates of transition metals (Zn, Cu, Co, and Ni) on the carrier was monitored using flame atomic absorption spectrometry (a novAA 300 atomic absorption spectrometer, Analytik Jena AG, Germany) and photometry methods. The quantitative determination of metals was carried out under conditions standard for each element by drying the samples, followed by decomposition of the residue with a mixture of acids.

The photometric determination was done using a PE-5300V (PromEcoLab, Russia) spectrophotometer at a wavelength of λ = 540 nm in a slightly acidic medium in the presence of sulfarsazen (plumbon) required for complexation with Zn^2+^ ions.

### Experiments on the Adsorption Desulfurization of Simulated Mixtures

These experiments were carried out in a laboratory flow-through device under atmospheric pressure at ambient temperature.^49^ Modified silica gel with a dense bulk layer (15 g) was placed in a cylindrical glass absorber. To estimate the effect of transition-metal (Zn, Cu, Co, and Ni) carboxylates on the extent of fuel purification from sulfides, we used individual hydrocarbons (hexane, heptane, “chemically pure”, ECROS, Russia). The simulated mixture of hydrocarbons with a composition similar to that of gasoline fractions consists of (mass %): *n*-alkanes—28.73, aromatic hydrocarbons—26.87, isoparaffins—22.50, cycloalkanes—21.49, and alkanes—0.41. The mixtures contained H_2_S, propyl- and isopropylthiols (98%, Alfa Aesar, USA).The simulated hydrocarbon mixture (*V* = 60 cm^3^) with an initial sulfur content of 50 or 100 ppm was fed to the absorber at a volume flow rate of 0.9 h^–1^. The total content of sulfur in the initial and purified simulated mixtures was determined using energy-dispersive X-ray (EDX) fluorescence spectrometry (ASE-1, Burevestnik, Russia, GOST R51947-2002) and cyclic voltammetry (IPC Pro, Russia). The experiments with different transition-metal carboxylates as silica gel modifiers were performed under the same conditions.

### Experimental Studies of the Adsorptive Process

The equilibrium adsorption of a sulfur-containing mixture on silica gel modified with metal (Zn, Cu, Co, and Ni) carboxylates was carried out in a temperature range from 0 to 80 °C. Modified silica gel (50 cm^3^) was weighed and placed in a conical flask with a ground stopper. The simulated hydrocarbon mixture [*V* = 75 cm^3^ (∼60 g)] with an initial total sulfur content of ca. 100 ppm was added to the weighed portion of the absorbent and then the flask was closed and placed into a thermostat for 3 h to attain adsorption equilibrium. The mixture was then decanted and analyzed. The total sulfur content in the initial and purified simulated mixtures was determined using EDX fluorescence spectrometry. The experiments were performed under the same conditions for all adsorbents.

### SEM and EDX Analyses

SEM and EDX analyses were performed using a Carl Zeiss NVision 40 high-resolution scanning electron microscope (Carl Zeiss, Germany) equipped with an Oxford Instruments X-MAX (80 nm^2^) detector operated at 20 kV acceleration voltage. For EDX analysis, the signal collection time was 100 s, and the data were processed using Oxford Instruments INCA software.

### Quantum Chemical Calculations

The density functional theory method [functional and basis: B3LYP/6-31++G(d,p)] and the Gaussian 09 program were used to simulate the adsorption of zinc carboxylates on a silica gel cluster. The calculations allowed us to estimate the thermal effect of the reactions of RSH with the carboxylates indicated above. The computations were performed with full optimization of the geometry of the structures.

## Results and Discussion

**Figure 1:**
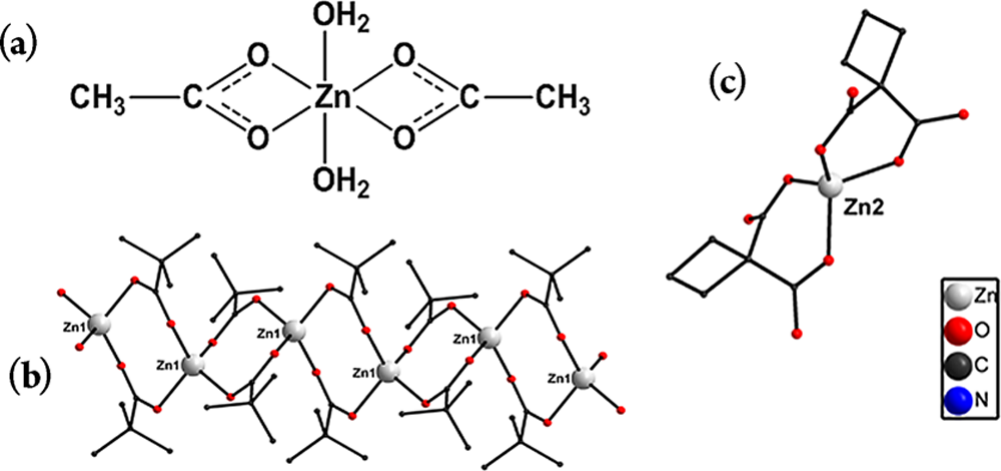
Zinc(II) acetate; (b) zinc(II) pivalate, a coordination polymer in which the zinc(II) atoms are bound pairwise by two anions of pivalic acid; and (c) zinc(II) “malonate” (cyclobutane-1,1-dicarboxylate).

The interest in carboxylate complexes of transition metals, especially those with the structure of coordination polymers, is due not only to the capacity of metal centers to take part in the oxidation process but also to the stability of these compounds in aqueous solutions (in the absence of hydrolysis) and an increased concentration of active metal centers on the surface of carriers. Transition-metal carboxylates can form the corresponding low-toxicity sulfides and RSH with H_2_S and RSH. It should be noted that the guiding principles of “green chemistry” proposed by P. T. Anastas and J. C. Warner include energy-saving and prevention of environmental pollution. In this study, we explore the ways to remove toxic H_2_S and low-molecular-weight RSH at room temperature and under atmospheric pressure. A significant drawback of many adsorption techniques is that they produce waste in the form of depleted catalytic agents. We used nontoxic silica gel as the carrier and transition-metal carboxylates that feature potential biological activity (Figure 1).

Using X-ray single-crystal analysis, we determined the structure of the hitherto unknown zinc cyclobutane-1,1-dicarboxylate (zinc “malonate”) complex, (NBu_4_)_4_[Zn(cbdc)_2_]_2_·3H_2_O. Compound 3 crystallizes in a triclinic space group *P*1̅ as two independent complex dianions [Zn(cbdc)_2_]^2–^, whose charges are compensated by four NBu^4+^ cations and three solvate water molecules. The zinc atom in [Zn(cbdc)_2_]^2+^ coordinates two cbdc-dianions in a bis-chelate manner (Zn–O 1.916(4)–1.943(3) Å, O–Zn–O 97.4(2)–125.5(2)°) to form a six-membered chelate ring, thus creating a tetrahedral ZnO_4_ chromophore. The solvate water molecules form H-bonds with the acceptor oxygen atoms of cbdc-dianions (O···O 2.839–2.874 Å, H···O 1.99–2.05 Å, and O–H–O 152.8–173.4°) in a crystal. The compounds 1–3 in question differ in solubility in aqueous and nonaqueous solvents. Zinc acetates and “malonates”, which are monomeric compounds, are soluble in water. Zinc pivalate, a polymeric compound, is insoluble in water but well soluble in organic solvents (e.g., THF). We also studied the carboxylates of other transition metals, namely, Co(CH_3_COO)_2_, [Cu_2_(Piv)_4_(Hpiv)_2_], [Co(Piv)_2_]_*n*_, and [Ni_9_(OH)_6_(Piv)_12_(Hpiv)_4_].

**Figure 2:**
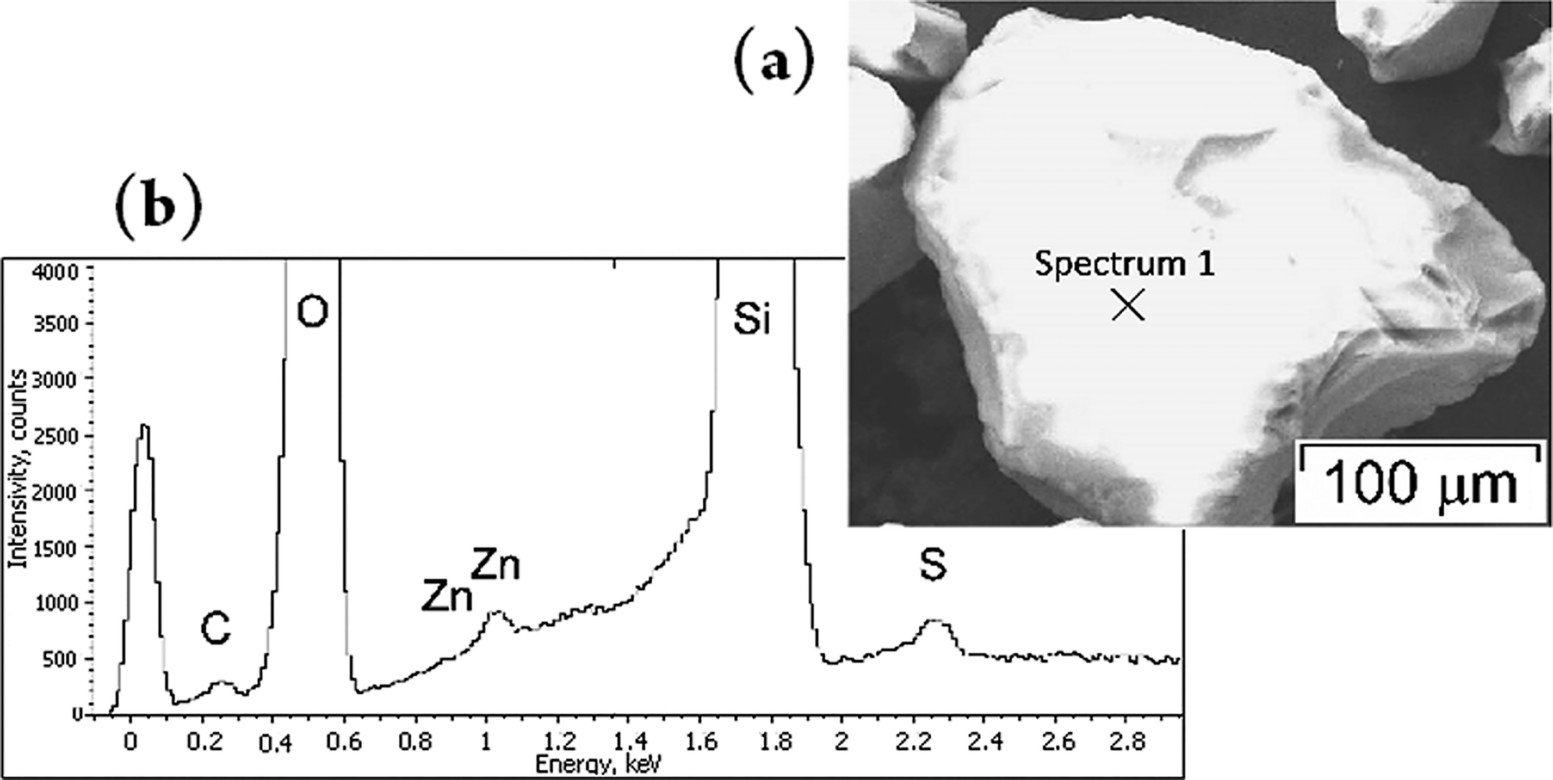
SEM image (a) and EDX spectrum (b) of the sample that indicates the presence of zinc and sulfur in porous silica.

Preliminary testing of the reactivity of complexes with respect to H_2_S and RSH by cyclic voltammetry showed that the sulfide oxidation peaks disappear instantaneously due to the reactions of sulfide with the complexes. As a result, the structure of the complexes disintegrates and sulfides [MS] or M(RS)_2_ precipitate, respectively. The energy dispersive X-ray spectrum of the surface of adsorbent particles [silica modified with polymeric zinc pivalate (2)] after desulfurization of the simulated hydrocarbon mixture clearly exhibits the presence of metal and sulfur ions (Figure 2a,b).

**Figure 3:**
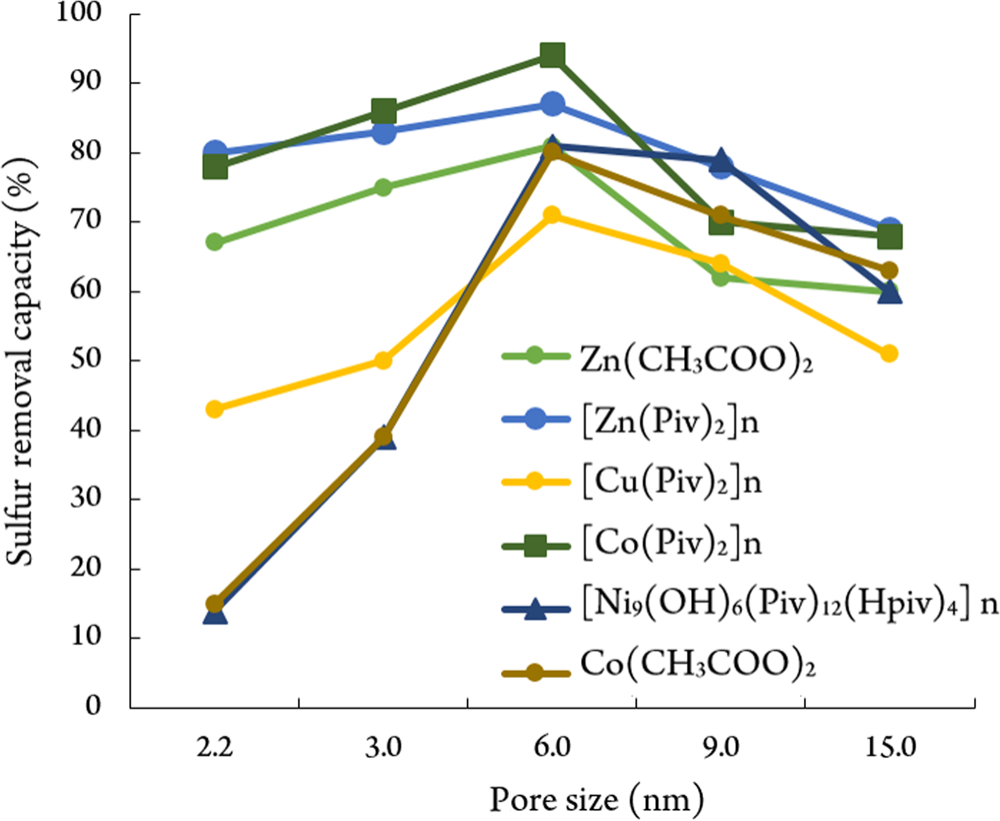
Extent of adsorption of 2-propanethiol as a function of the pore size of modified silica gel.

It is known that silica gel has a well-developed surface and may have pores of different sizes that depend on the method of its preparation. In this work, we chose silica gel with optimal dimensions of pores that provide the maximal extent of adsorption of acidic sulfur-containing components from simulated hydrocarbon mixtures after modifying the carrier surface with metal carboxylates (1 mass %) (Figure 3).

The data presented in Figure 3 show that the maximum extent of adsorption of acidic sulfur-containing components is observed on the modified silica gel with a pore size of 6 nm. Subsequently, we used commercial, easily available, and relatively inexpensive silica gel of Alfa Aesar brand whose specific surface area is 480–540 m^2^/g, the total volume of pores is 0.75 cm^3^/g, and the average pore diameter is 6 nm.

**Figure 4:**
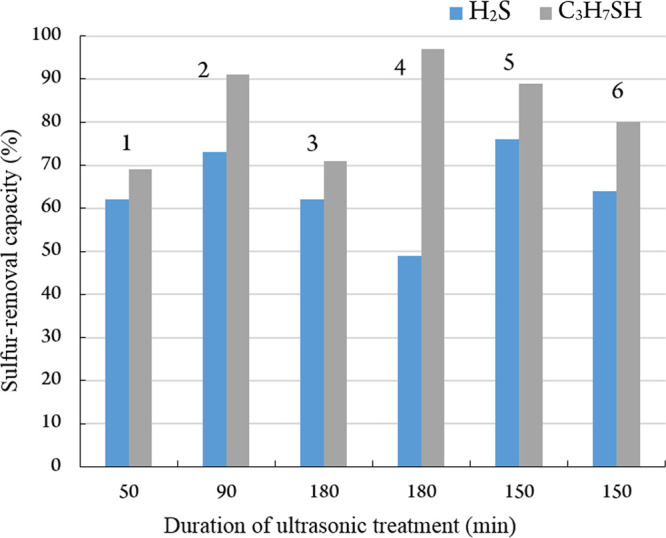
Adsorption of H_2_S and RSH (initial concentration, 100 ppm) by silica gel modified with metal complexes (1 mass %) as a function of the duration of ultrasonic treatment. 1—Zn(CH_3_COO)_2_, 2—[Zn(Piv)_2_]_*n*_, 3—[Cu(Piv)_2_]_*n*_, 4—[Co(Piv)_2_]_*n*_, 5—[Ni_9_(OH)_6_(Piv)_12_(Hpiv)_4_]_*n*_, and 6—Co(CH_3_COO)_2_.

The two-stage treatment (impregnation and drying) of the carrier with solutions of carboxylates requires a rather long time (48 h). To save time and enhance the efficient retention of complexes on the silica gel surface, we used an energy-saving process, namely, ultrasonic treatment, in adsorbent modification (Figure 4).

**Figure 5:**
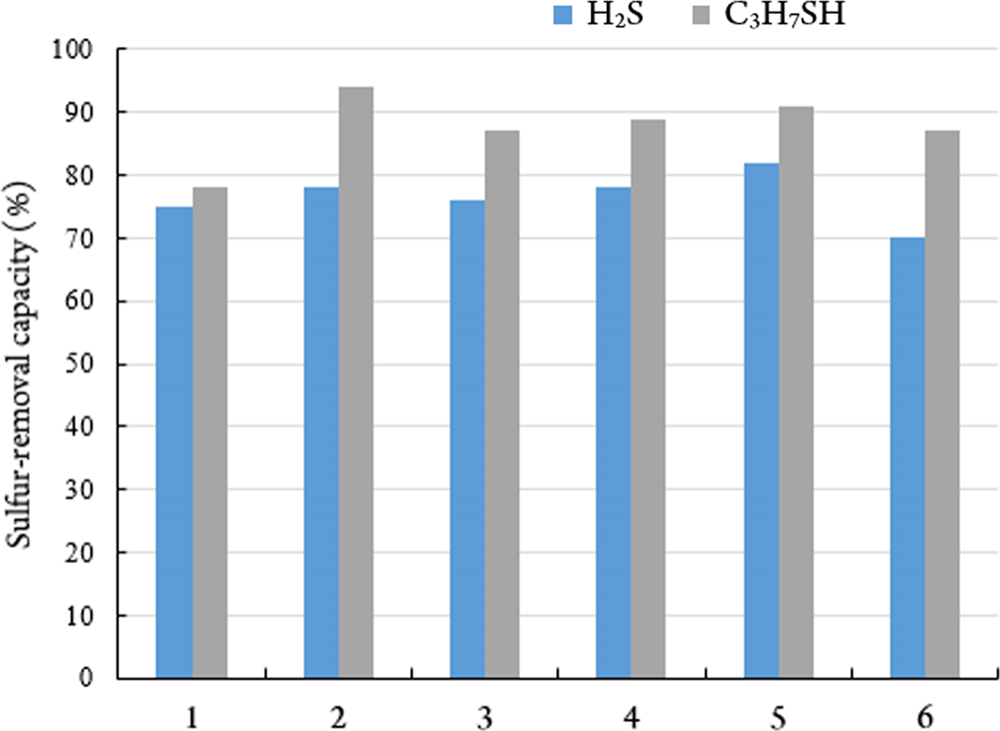
Extent of absorption of H_2_S and 2-propanethiol by complexes deposited on silica gel (1 mass %) upon three-stage adsorption. 1—Zn(CH_3_COO)_2_, 2—[Zn(Piv)_2_]n, 3—[Cu(Piv)_2_]n, 4—[Co(Piv)_2_]n, 5—[Ni_9_(OH)_6_(Piv)_12_(Hpiv)_4_]_*n*_, and 6—Co(CH_3_COO)_2_.

It follows from Figure 4 that zinc(II) pivalate features the highest efficiency with respect to both sulfur-containing reagents, but the maximum extent of purification from RSH is observed if cobalt pivalate is used. A comparison of complexes that contain acetate and pivalate anions shows that their adsorption activity is fairly equivalent, but RSH are better adsorbed by polymeric zinc pivalate 2 (Figure 5).

A comparison of complexes that contain acetate and pivalate anions shows that their adsorption activity is fairly equivalent, but RSH are better adsorbed by polymeric zinc pivalate 2 (Figure 5). The results displayed indicate a rather high efficiency for all framework polymeric structures of Zn, Cu, Co, and Ni pivalates. The best results for the removal of H_2_S were obtained for the nine nuclear-mixed nickel(II) complex, whereas polymeric zinc(II) pivalate is recommended for the adsorption of RSH. Experiments on the adsorption of H_2_S by silica gel modified with polymeric zinc acetate and pivalate and monomeric zinc “malonate” show the advantage of zinc pivalate.

The efficiency of adsorption of sulfides from simulated mixtures on modified silica gel can be estimated by studying the adsorption in an equilibrium mode. Studies in this mode ensure the determination of the maximum amount of the adsorbed compound. If a compound being adsorbed remains in contact with the adsorbing agent for a sufficiently long time, the state of the system is close to equilibrium. The degree of purification attained during the establishment of adsorption equilibrium corresponds to the maximum adsorption efficiency of sulfur-containing compounds from this mixture for a specific adsorbent.

Table 2 displays the values of the efficiency of 2-propanethiol adsorption from the simulated mixture on silica gel modified with metal carboxylates (Zn, Cu, Co, and Ni). The adsorption capability was calculated using eq 2
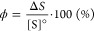
2where Δ[S] = [S]° – [S] (mg/kg); here, [S]° is the concentration of total sulfur in the unpurified raw material (mg/kg), [S] is the residual concentration of total sulfur in the purified raw material (mg/kg), and Δ[S] is the concentration of total adsorbed sulfur [with respect to the mass of the raw material, i.e., Δ[S] = *m*(*S*)/*M* (mg/kg), where *M* is the mass of the raw material and *m*(*S*) is the total mass of sulfur (mg)].

**Table 2:** Parameters for the Adsorption of Sulfur-Containing Simulated Mixture on Silica Gel Modified with Metal Carboxylates

	Zn(CH_3_COO)_2_		[Zn(Piv)_2_]*n*		[Cu(Piv)_2_]*n*	[Co(Piv)_2_]*n*		[Ni_9_(OH)_6_(Piv)_12_(Hpiv)_4_]		
temperature, °C	φ, %	*a*, mg/g	φ, %	*a*, mg/g	φ, %	*a*, mg/g	φ, %	a, mg/g	φ, %	*a*, mg/g
20	21	0.045	34	0.11	25	0.07	20	0.05	16	0.03
40	28	0.055	69	0.23	34	0.07	40	0.10	39	0.08
65	34	0.065	92	0.26	46	0.12	59	0.15	87	0.16
80	20	0.036	51	0.15	38	0.09	30	0.08	67	0.14

The amount of adsorbed substance (*a*) was calculated using eq 3
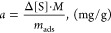
3where *m*_ads_ is the mass of the adsorbent, g.

Experimental data on equilibrium adsorption on silica gel modified with zinc acetate and metal pivalates show that zinc pivalate is effective against RSH. The results of quantum mechanical calculations that simulate some possible paths of the reactions of RSH with zinc carboxylates are presented in Table 3.

**Table 3:** Energy Effects (Δ*E*) in the Reactions of RSH C1–C3 with Various Zinc Carboxylates

reaction scheme	Δ*E*, kJ/mol
CH_3_SH + ZnPiv_2_ = HPiv + PivCH_3_ + ZnS (Zn_6_S_6_ cluster)	6.5
C_2_H_5_SH + ZnPiv_2_ = HPiv + PivC_2_H_5_ + ZnS (Zn_6_S_6_ cluster)	–1.3
*n*-C_3_H_7_SH + ZnPiv_2_ = HPiv + PivC_3_H_7_ + ZnS (Zn_6_S_6_ cluster)	–0.4
2CH_3_SH + ZnPiv_2_ = 2HPiv + Zn(SCH_3_)_2_	15.4
2C_2_H_5_SH + ZnPiv_2_ = 2HPiv + Zn(SC_2_H_5_)_2_	16.5
2*n*-C_3_H_7_SH + ZnPiv_2_ = 2HPiv + Zn(SC_3_H_7_)_2_	16.6
CH_3_SH + ZnAc_2_ = AcH + AcCH_3_ + ZnS (Zn_6_S_6_ cluster)	1.4
C_2_H_5_SH + ZnAc_2_ = AcH + AcC_2_H_5_ + ZnS (Zn_6_S_6_ cluster)	–5.5
*n*-C_3_H_7_SH + ZnAc_2_ = AcH + AcC_3_H_7_ + ZnS (Zn_6_S_6_ cluster)	–4.6
CH_3_SH + Zn(cbdc)_2_^2–^ = HOOC-cb-COOCH_3_ + ^–^OOC-cb-COO^–^ + ZnS (Zn_6_S_6_ cluster)	349.9
C_2_H_5_SH + Zn(cbdc)_2_^2–^ = HOOC-cb-COOC_2_H_5_ + ^–^OOC-cb-COO^–^ + ZnS (Zn_6_S_6_ cluster)	342.7
*n*-C_3_H_7_SH + Zn(cbdc)_2_^2–^ = HOOC-cb-COOC_3_H_7_ + ^–^OOC-cb-COO^–^ + ZnS (Zn_6_S_6_ cluster)	343.3

A fragment of ZnS structure was simulated using Zn_*n*_S_*n*_ clusters (*n* = 1, 2, 4, and 6). Calculations show that as *n* grows (i.e., the size of the model cluster increases), the value of Δ*E* decreases.

It may be concluded from the calculation results presented in Table 3 that the most probable channel of desulfurization is the reaction of RSH with zinc pivalate to give ZnS, since these reactions are characterized by lower Δ*E* values compared with the alternative channel of demercaptanization to give Zn(SR)_2_ products.

The nature of the hydrocarbon radical in the RSH does not significantly affect the energy balance of the model reactions under consideration: the corresponding Δ*E* values in the case of methanethiol, ethanethiol, and propanethiol are fairly close to each other.

According to the data shown in Table 3, the Δ*E* values for the model desulfurization reactions that involve zinc “malonate” are significantly larger than those for similar reactions with zinc pivalate. Thus, in terms of the energy parameters of the model reaction, zinc “malonate” should exhibit a significantly smaller activity in desulfurization processes compared to that of pivalate, which agrees with the experimental data. The reason for this difference between the activities of zinc carboxylates is apparently that zinc “malonate” has a more stable structure than pivalate and is less prone to “decomposition”.

To identify the effect of the anion nature on the adsorption of complexes on silica gel, calculations were performed using zinc salts as an example. We obtained optimized geometric parameters of the models of adsorption complexes formed between zinc pivalate, acetate, “malonate”, and silica gel clusters. The adsorption energies calculated for zinc pivalate, acetate, and “malonate” were −97.9, −99.2, and −165.0 kJ/mol, respectively.

The adsorption energy in the case of zinc “malonate” is more negative than that of pivalate or acetate. This implies that zinc “malonate” forms a stronger adsorption complex and may be retained on the silica gel surface more strongly, which may additionally explain its less pronounced reaction capacity toward RSH.

In the next stage of this study, the algorithm for adsorption removal of acidic sulfur-containing components that we developed was applied to model analogues of gasoline fractions. The mixtures contained H_2_S, propyl- and isopropylthiols (the total amount of sulfur was no larger than 50 ppm). The group composition (mass %) of the simulated mixture was as follows: *n*-alkanes—28.73, aromatic hydrocarbons—26.87, isoparaffins—22.50, cycloalkanes—21.49, and alkanes—0.41. Experiments on three-stage adsorption of sulfur-containing components in the model gasoline fraction by silica gel modified with zinc and cobalt pivalates [Zn(Piv)_2_/Co(Piv)_2_] have shown that at stage I, the total content of sulfur is reduced by 48% and, at stage II, additional purification by 29.6% occurs. The extent of additional purification at stage III was 74% if Zn(Piv)_2_ was used (Table 4).

**Table 4:** Results of Multistage Adsorption of Sulfur-Containing Components in the Model Gasoline Fraction on Silica Gel Modified with Zinc or Cobalt Pivalates [Zn(Piv)_2_/Co(Piv)_2_]

parameter	raw material	I	II	III
total sulfur, ppm	52/49	27/23	19/16	5/3
extent of purification φ, %		48/53	63/67	90/94

To identify the synergistic effect of a combination of complexes, we studied the double system of zinc and cobalt pivalates (1:1) (Table 5).

**Table 5:** Results of Multistage Adsorption of Sulfides in the Model Gasoline Fraction with an Initial Boiling Point of 64 °C on Silica Gel Modified with Pivalates of Zinc or Cobalt [Zn(Piv)_2_/Co(Piv)_2_]

parameter	raw material	I	II	III
total sulfur, ppm	103/102	60/34	18/20	4/5
extent of purification φ, %	–/–	42/67	83/80	96/95

physicochemical and operational parameters of gasoline fraction								
octane number				fractional composition, °C				
research method	motor method	density at 20 °C, kg/m^3^	saturated vapor pressure, kPa	initial boiling point	10% vol	50% vol	90% vol	end boiling point
73	70	703	13	64	75	96	139	161

If the surface of silica gel is concurrently modified with zinc and cobalt pivalates, the content of toxic sulfides in gasoline fractions decreases to 3–5 ppm, indicating that an insignificant synergistic effect exists.

## Conclusions

This study intentionally focuses on the use of zinc complexes to modify silica as a carrier. In this case, upon adsorption of toxic acidic sulfur-containing components, the complexes are converted to compounds that are not only safe but are also biologically active as they exhibit antimite and antifungal effects (ZnS) and antioxidant effects [Zn(SR)_2_]. Silica gel treated with a coordination polymer, zinc pivalate, after its use for adsorption of sulfur-containing admixtures, may be recommended as an agent for treating the running track and sports grounds, since the resulting ZnS and RSH that are produced in insignificant amounts manifest anti-mite, antifungal,^50,51^ and antioxidant^52^ effects. Zinc pivalates can be synthesized in commercial amounts, and the final product enables the removal of acidic sulfur-containing components without damaging the environment in small-scale gas refining plants where state-of-the art hydrotreatment is not profitable.

## Abbreviations

OPC: oxygen-functionalized porous carbon
EDTA: ethylenediaminetetraacetic acid
SEM: scanning electron microscopy
TEM: transmission electron microscopy
Piv: pivalate anion, pivaloyl
Ac: acetate anion
cb: cyclobutyl
cbdc: cyclobutane dicarboxylate
